# Systematic review of the rotavirus infection burden in the WHO-EMRO region

**DOI:** 10.1080/21645515.2019.1603984

**Published:** 2019-05-17

**Authors:** Selim Badur, Serdar Öztürk, Priya Pereira, Mohammad AbdelGhany, Mansour Khalaf, Youness Lagoubi, Onur Ozudogru, Kashif Hanif, Debasish Saha

**Affiliations:** a MENA, Medical & Clinical Emerging Markets, GSK, Istanbul, Turkey; b Global Medical Affairs Rota/MMRV, GSK, Wavre, Belgium; c EM Central Vaccines Medical/Clinical, GSK, Cairo, Egypt; d EM Central Vaccines Medical/Clinical, GSK, Jeddah, Saudi Arabia; e EM Central Vaccines Medical/Clinical, GSK, Casablanca, Morocco; f EM Central Vaccines Medical/Clinical, GSK, Dubai, United Arab Emirates; g EM Central Vaccines Medical/Clinical, GSK, Karachi, Pakistan; h Epidemiology/Health Economics EM, GSK, Wavre, Belgium

**Keywords:** Rotavirus, gastroenteritis, children, Eastern Mediterranean region, burden, incidence

## Abstract

Rotavirus gastroenteritis imposes a heavy burden on low- and middle-income countries. The World Health Organization defines the Eastern Mediterranean region (WHO-EMRO) as a diverse area in terms of socioeconomic status and health indicators. Rotavirus vaccination has been introduced, at least partially, in 19 out of the 22 EM countries; however, vaccine coverage remains low, and data on rotavirus disease burden is scarce.

Available data on rotavirus prevalence, seasonality, vaccination status, and genotype evolution was systematically compiled following a literature review that identified 165 relevant WHO-EMRO epidemiology studies published between 1990 and 2017.

Although the infectious agents responsible for acute gastroenteritis vary over time, rotavirus remained the leading cause of acute gastroenteritis in children, as seen in 76.3% of reviewed publications. Younger children (<2 years old) were at higher risk and thus increased vaccination coverage and surveillance systems are required to reduce the rotavirus gastroenteritis burden in WHO-EMRO countries.

## Introduction

Rotavirus (RV) is the leading cause of diarrheal morbidity and mortality in young children worldwide and causes severe acute gastroenteritis (AGE) requiring hospitalization, and if dehydration is not treated in time leads to mortality especially in the developing world.^1^ By the age of five years, nearly every child will have had an episode of RV gastroenteritis (RVGE), one in five of them will visit a clinic, one in 65 will be hospitalized, and approximately 1 out of 293 will eventually have a fatal outcome.^2,3^ According to a report, more than 90% of RVGE deaths in 2013 occurred in 72 low-income and low-middle-income countries.^4^ Implementation of an RV vaccination in national immunization programs (NIPs) reduces the RV disease burden substantially.^5^ Countries need to have adequate knowledge and information on local burden, trends, and age distribution of disease to help decision-makers consider the introduction of an RV vaccine as part of their immunization programs.^6^

**Figure 1.**
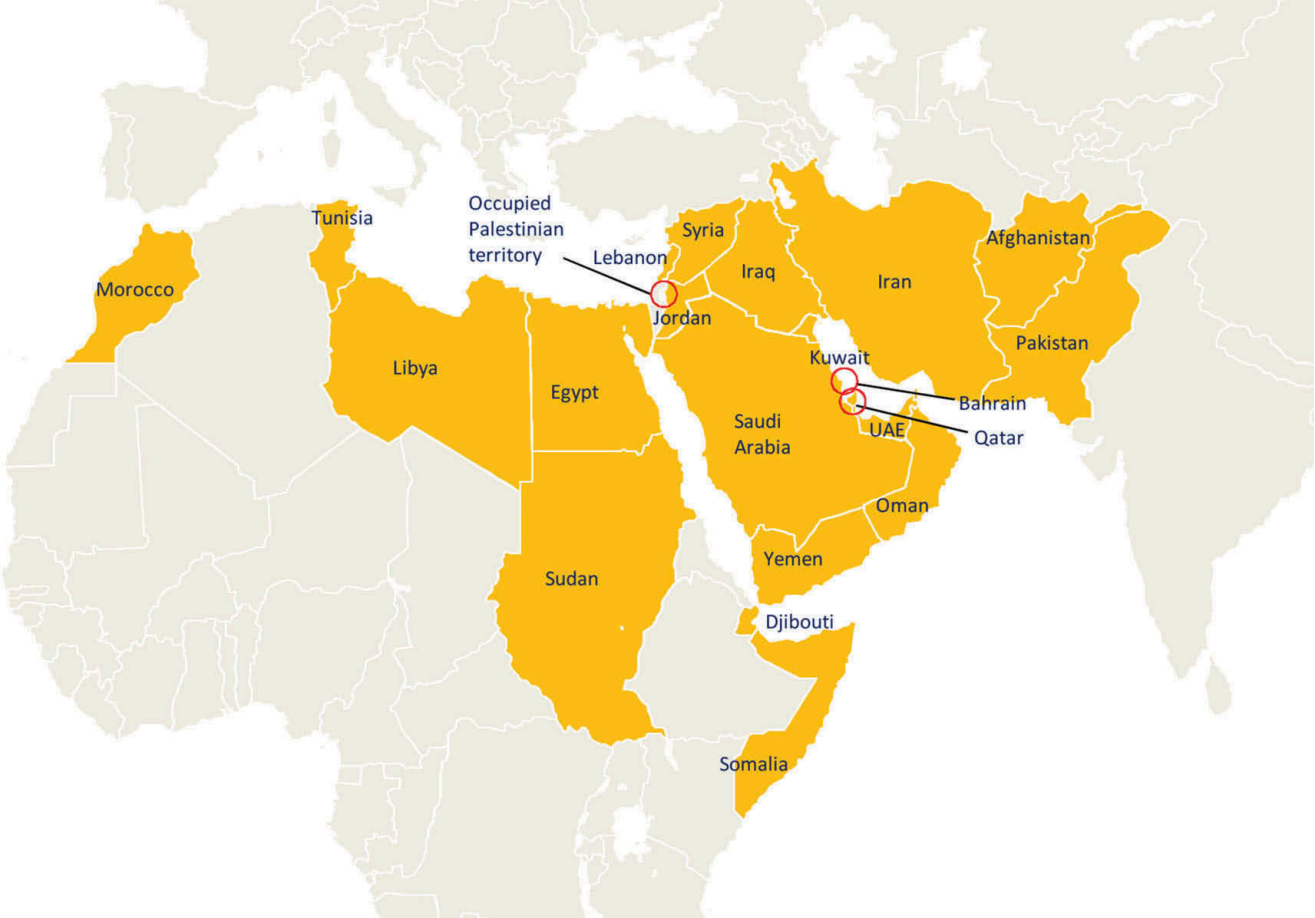
WHO-EMRO region countries (i.e. Afghanistan, Bahrain, Djibouti, Egypt, Iran (Islamic Republic of), Iraq, Jordan, Kuwait, Lebanon, Libya, Morocco, Occupied Palestine Territory, Oman, Pakistan, Qatar, Somalia, Sudan, Syrian Arab Republic, Tunisia, United Arab Emirates (UAE), and Yemen) included in the study. UAE: United Arab Emirates.

The Eastern Mediterranean region, as defined by the World Health Organization (WHO-EMRO region, Figure 1), is a geographically and socioeconomic diverse area with varying health indicators. At present, the region has a population of over 656 million (81.4 million (12.4% of the total population) – is under 5 years of age).^7^ RV-associated mortality and morbidity also vary considerably in the region: the annual morbidity rates among children under five years of age ranged from 0 to 112/100,000 with an average mortality rate of 39/10,000 per year.^5,8^ Low-income countries (e.g. Afghanistan, Pakistan, Sudan, Yemen, and Somalia) had a higher mortality rate due to RVGE compared with countries where the per capita income was high (e.g. Saudi Arabia and Kuwait). However, the overall hospital and health center visits due to RVGE among children under five years of age was similar in both high- and low-income WHO-EMRO countries.^5,8,9^

**Figure:**
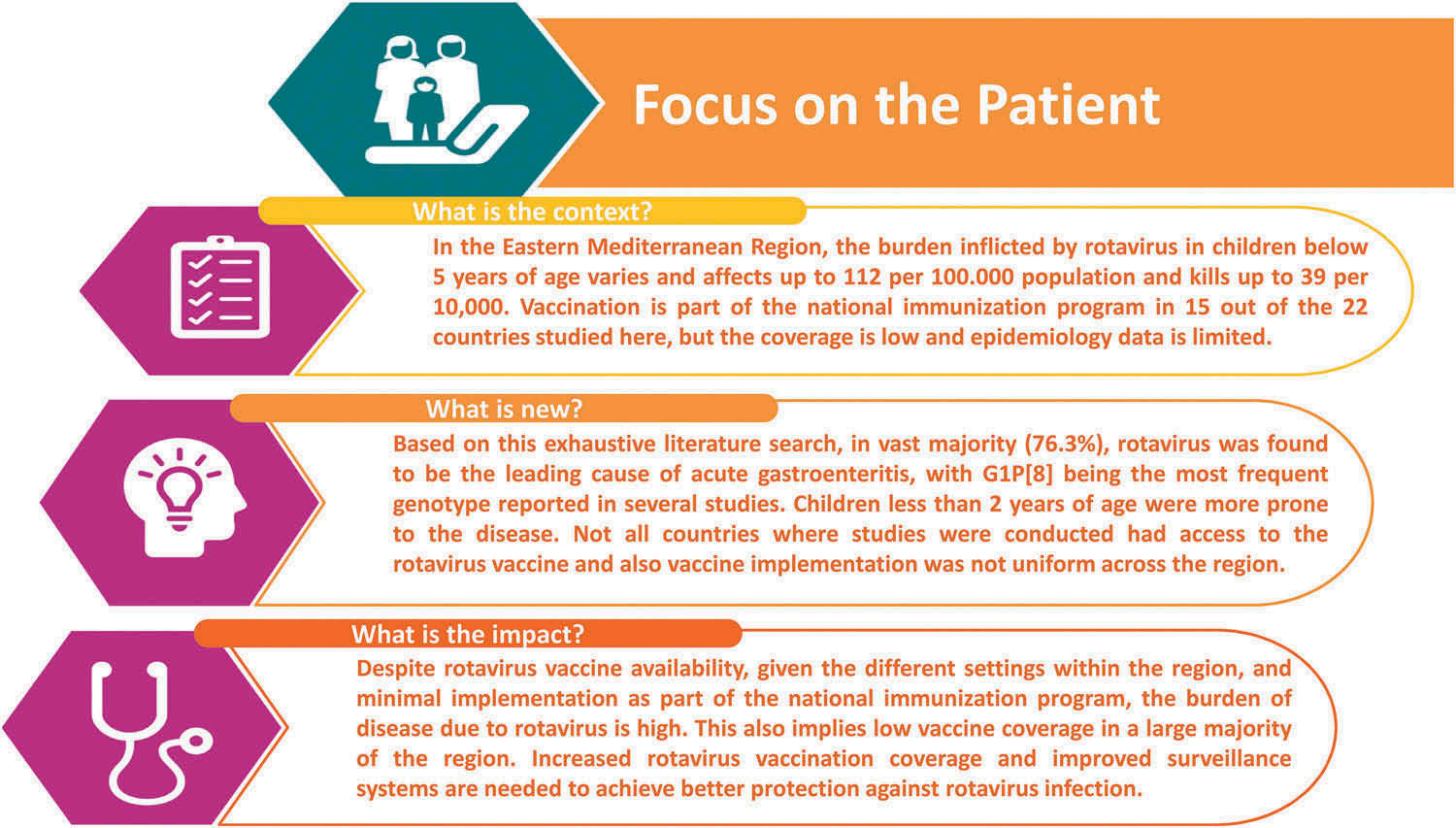

An RV vaccine has been introduced as part of the NIPs in 15 out of the 22 countries (albeit partially in Pakistan and Palestine) in the WHO-EMRO region. Vaccine coverage is predominantly suboptimal, with the exception of Saudi Arabia, with 97% vaccine coverage, and burden of disease data are still scarce.^1,6,10^ Furthermore, a lack of surveillance systems prevents decision makers from understanding the magnitude of the problem and the need for RVGE prevention through vaccination.^11^

The purpose of this literature review is to assess the burden of RVGE in the pediatric population and to summarise the current status of recommendations for RV vaccination across the WHO-EMRO region. Moreover, this review will also be useful as a baseline for post-vaccination surveillance for both AGE and RVGE.

## Results

**Figure 2.**
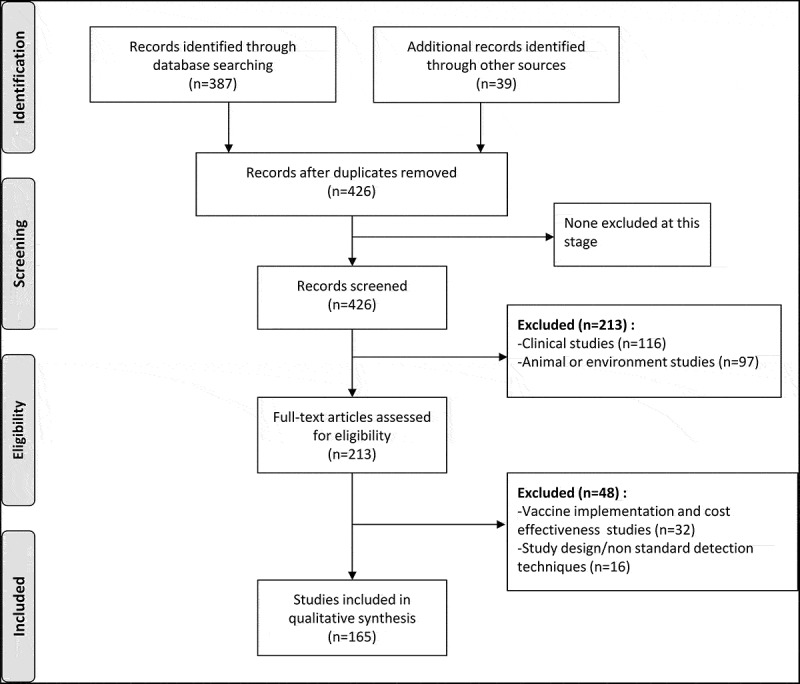
Literature search strategy (limited to articles published between 1990 and 2017 with abstracts in English or French) using the keywords “rotavirus” and “[country name]”.

Four hundred and twenty-six articles were identified through the search process as of 5^th^ December 2017. Two hundred and thirteen publications were excluded and 213 were included for full-text review. One hundred and sixty-five publications were included in the qualitative assessment (Figure 2). The countries and number of associated references from which published data were obtained are: Afghanistan (1),^12^ Bahrain (3), ^13–15^ Egypt (17),^16–32^ Iran (37),^33–68^ Iraq (3),^41,69,70^ Jordan (9),^71–79^ Kuwait (2),^80,81^ Lebanon (3),^82–84^ Libya (5),^85–89^ Morocco (9),^90–98^ Oman (4),^99–102^ Palestine (2),^103,104^ Pakistan (15),^105–119^ Qatar (1),^120^ Saudi Arabia (21),^121–141^ Somalia (2),^142,143^ Sudan (4),^144–147^ Tunisia (20),^148–167^ United Arab Emirates (UAE) (3),^168–170^ and Yemen (4).^171–174^ No study related to Djibouti could be retrieved.

Details on study subjects, dates, settings and calculated RV prevalence from the studies across the region is shown in Tables S1-S3. Table S1 presents data from the North African countries (Egypt, Morocco, Tunisia, Somalia, Sudan, and Libya), Table S2 includes the Middle Eastern countries (Saudi Arabia, Lebanon, Bahrain, Oman, Palestine, Qatar, UAE, Yemen, Jordan, Iraq, and Kuwait) and Table S3 shows data from the Asian countries (Iran, Pakistan, and Afghanistan). A total of 113,959 cases of confirmed RVGE are reported in this review, with the highest number from Pakistan (n = 13,546) and the lowest from Somalia (n = 213).

### Prevalence of RVGE

The prevalence of RV infection from different studies across the three regions (Nord Africa, Middle East, and Asia) is shown in Table 1.

**Table 1.** Prevalence of RVGE among AGE cases in studies from 1990 to 2017, per country.

Region	Country (number of studies)	References number	Total number of patients	Prevalence range	Prevalence mean	Prevalence median
North Africa	Egypt (17)	16–32	5,367	11–76.9	35.9	33.6
	Morocco (9)	90–98	9,044	17.2–44	33.8	37.5
	Tunisia (20)	148–167	10,094	6.1–33.9	23.0	23.7
	Sudan (4)	144–147	12,432	11.7–36	21.4	19.0
	Libya (5)	85–89	1,914	13.4–58	38.6	33.0
Middle East	Saudi Arabia (21)	121–141	12,713	3.9–65.5	28.3	26.9
	Lebanon (3)	82–84	1,980	27.9–48.1	35.4	30.3
	Bahrain (3)	13–15	1,590	13.9–44.8	26.5	20.8
	Oman (4)	99–102	10,031	31.3–57.4	46.6	48.9
	UAE (3)	168–170	4,191	21.4–50.3	32.1	24.7
	Yemen (4)	171–174	9,666	14.1–45.2	30.4	31.1
	Jordan (9)	71–79	4,709	18.9–49.5	34.0	35.0
	Iraq (3)	41, 69, 70	1,320	18.5–40.4	31.9	36.9
Asia	Iran (37)	33–68	14,204	15.3–70.2	34.9	29.5
	Pakistan (15)	105–119	13,546	8.2–65.9	31.4	29.2

Countries with ≥3 studies are presented in this table (i.e. data from Somalia, Afghanistan, Kuwait, Palestine, Qatar are not included). This table includes only data from children >5 years old, and studies reporting prevalence data with the standard EIA technique. The patient number includes control cases but only associated to prevalence endpoint (i.e. patient count with other endpoints, eg. sequencing, were excluded). For studies showing results evolution over time, only the most recent data was taken into account for mean and median calculation. For studies describing inpatient and outpatient RV prevalence, the individual values per study were calculated as total RV cases among all patients (inpatients+outpatients) divided by total number of patients (inpatients+outpatients).

**Figure 3.**
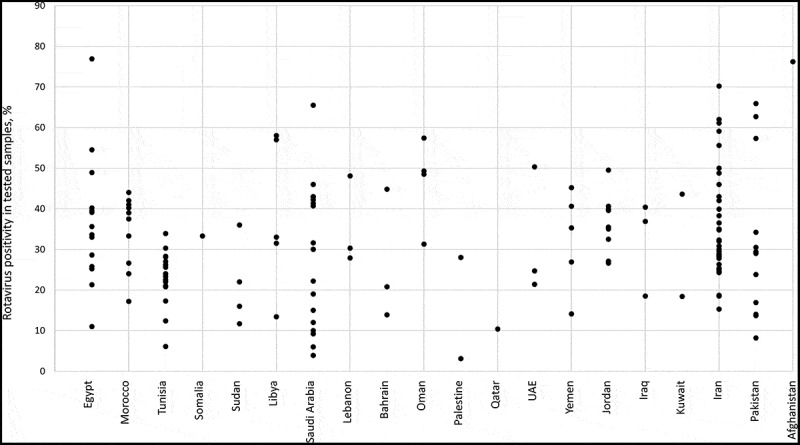
Prevalence of RVGE among cases of AGE in individual studies per country. The overall incidence was reported for studies detailing prevalence over several years, except when the study was comparing pre- and post-vaccination periods. In this case, the latest value was retained, as more actual.

Among the 165 articles included in this review, 152 (92.1%) reported RV prevalence data. The mean RV prevalence among AGE cases in the EMRO countries with more than two studies was between 21.4% (Sudan) and 46.6% (Oman) and the median was between 19.0% and 48.9%. The prevalence of RV in AGE cases was over 20% in 121 (79.6%) of them, with the highest reported rates observed in Egypt (76.9%), Afghanistan (76.2%) and Iran (70.2%). (Table 1, Figure 3)^66^ There were differences in RV isolation rates among multiple studies conducted within the same country, e.g. Pakistan (8.2% to 65.9%), Saudi Arabia (3.9% to 65.5%) and Egypt (range: 11% to 76.9%). However, in other countries, prevalence appeared to vary less, as seen by a narrower distribution in Figure 3 for certain countries including Morocco (17.2% to 44%), Tunisia (6.1% to 33.9%), or Jordan (26.6% to 49.5%)

There were also important differences in inpatient and outpatient RV prevalence. For example, in Egypt, in the 2011–2012 period, and using the same technique, the RV prevalence was 29.9% for outpatients and 43.9% for inpatients;^29^ in Tunisia, this prevalence was 18.5% and 24.9%,^166^ and in Bahrain 6% and 27.4%, respectively.^13^

### Changes in the detection of RVGE over time

In Jordan, the RVGE detection rates among AGE cases were fairly constant over a ten-year period (25% in 1995, 35.2% in 2002, 24.7% in 2003, and 25.8% in 2004).^76^ On the other hand, the proportion of RVGE cases appears to have increased over time in Pakistan; as the RVGE rates were ranging from 8.2% to 29% in the earlier years (1985–1996),^105,107,116^ while in the more recent years (2007–2014) the reported rates were ranging from 23.8 to 65.9.^110–115,118,119^ This can be due to the difference of sensibility of EIA used during these two different periods, or to the choice and characteristics of patients investigated.

A reduction in RVGE occurrence is clearly evident after the introduction of RV vaccination in some countries. For instance, there was a 41.5% reduction in RV detection rate in the post-vaccination period compared to the pre-vaccination period in Morocco.^96^ In Yemen there was a steady decline in RV detection following vaccine introduction; the detection rates during the pre-vaccination period was 43.8% and 37.4% in 2009 and 2010, respectively, but declined during the post-vaccination period to 17.9% in 2013 and to 10.5% in 2014.^174^ In the same study, a 75.9% reduction in hospitalization due to RV diarrhea was reported over five years (2009: 43.8%; 2014: 10.5%). Banajeh *et al*.^173^ also showed that in Yemen, after vaccination had started, the prevalence of RVGE decreased by 48% and the hospitalization rate due to RV diarrhea declined from 42.9% during the pre-vaccination period (2007/2011) to 18.5% in the post-vaccination period (2013/2014). A similar pattern was observed in Saudi Arabia, where the RVGE prevalence was reduced from 12% to 46% to 9.2% over a three-year period following the introduction of the RV vaccine in 2013.^141^

### Concomitant enteropathogens

About one fourth (38 of 165) of the reviewed publications concomitantly reported isolation of pathogens other than RV. However, RV was most frequently responsible for AGE cases in 29/38 (76.3%), second most frequent in 5/38 (13.2%) and third most frequent in 3/38 (7.9%) of the studies. Norovirus, Astrovirus, and Adenovirus were other common viral pathogens detected in AGE cases (Table 2).

**Table 2.** Distribution of enteropathogens isolated in AGE cases in the WHO-EMRO region.

Country	Study	Nb of cases	1^st^ pathogen (%)	2^nd^ pathogen (%)	3^th^ pathogen (%)
Iran	Yahyapour *et al*., 2008^65^	208	RV (61.1)	Adenovirus (2.9)	Astrovirus (2.4)
	Hamkar *et al*., 2010^43^	400	RV (62.0)	Adenovirus (2.3)	Astrovirus (3.0)
	Ataei-Pirkooh *et al*., 2011^67^	100	RV (43.0)	*E. coli* (20.0)	*G. lamblia* (6.0)
	Motamedifar *et al*., 2013^50^	827	RV (42.0)	Adenovirus (88.7)	
	Najafi *et al*., 2013^51^	375	RV (24.3)	Norovirus (12.5)	Adenovirus (5.1)
	Shokrollahi *et al*., 2014^52^	80	RV (48.8)	Parechovirus (23.2)	Adenovirus (20.0)
	Sharifi-Rad *et al*., 2015*^56^*^.^	82	RV (70.2)	Adenovirus (20.3)	Norovirus (9.5)
Egypt	Pazzaglia *et al*., 1993^17^	880	RV (28.6)	*G. lamblia* (21.3)	*Campylobacter* (16.8)
	El-Mohamady *et al*., 2006^21^	253	RV (21.3)	ETEC (10.8)	*Cryptosporidium* (10.7)
	Kamel *et al*., 2009^23^	230	RV (33.6)	Norovirus (13.7)	
	El-Shabrawi *et al*., 2015^31^	365	RV (10.7)	ETEC (7.0)	*Cryptosporidium* (3.9)
Pakistan	Mubashir *et al*., 1990^105^	402	EPEC (32.8)	ETEC (14.2)	RV (8.2)
	Huilan *et al*., 1991^106^	758	RV (14.0)	Adenovirus (6.0)	*G. lamblia* (3.0)
	Alam *et al*., 2015^114^	563	RV (65.9)	Parechovirus (21.0)	Norovirus (19.5)
Tunisia	Moalla *et al*., 1994^149^	170	RV (12.4)	*G. lamblia* (5.9)	*Campylobacter* (4.7)
	Fodha *et al*., 2006^151^	638	RV (20.8)	Astrovirus (7.1)	Adenovirus (5.5)
	Al-Gallas *et al*., 2007^152^	115	ETEC (32.3)	Adenovirus (10.4)	RV (6.1)
	Sdiri-Loulizi *et al*., 2011^158^	632	RV (22.5)	Norovirus (17.4)	Astrovirus (4.1)
	Ben Salem-Ben Nejma *et al*., 2014^163^	124	RV (33.9)	EAEC (23.4)	ETEC (21.0)
Lebanon	Al-Ali *et al*., 2011^82^	79	RV (48.1)	Norovirus (6.3)	
Libya	Rahouma *et al*., 2011^86^	239	Norovirus (15.5)	RV (13.4)	DEC (11.2)
	Abugalia *et al*., 2011^88^	520	RV (31.5)	Norovirus (17.5)	
Afganistan	Elyan *et al*., 2014^12^	432	RV (76.2)	*Cryptosporidium* (14.1)	*G. lamblia* (5.1)
Bahrain	Ismaeel *et al*., 2002^14^	653	RV (13.9)	*Salmonella* (7.0)	*Shigella* (4.0)
Oman	Aithala *et al*., 1996^99^	217	RV (31.3)	*G. lamblia* (10.6)	*E. coli* (9.7)
Palestine	Abu-Elamreen *et al*., 2008^103^	150	RV (28.0)	*E. histolitica* (15.3)	*Shigella* (6.0)
	Laham *et al*., 2015^104^	150	*E. histolitica* (28.0)	*G. lamblia* (26.7)	RV (3.1)
Qatar	Al-Thani *et al*., 2013^120^	288	Norovirus (28.5)	RV (10.4)	Adenovirus (6.3)
Sudan	Elhag *et al*., 2015^144^	710	RV (11.7)	Adenovirus (2.3)	
	Saeed *et al*., 2015^147^	437	DEC (48.3)	RV (22.0)	*G. intestinalis* (10.8)
Jordan	Battikhi, 2002^74^	1,400	RV (26.6)	*Salmonella* (10.7)	EPEC (3.9)
	Nimri *et al*., 2004^75^	143	*Enterobacteriaceae* (46.2)	RV (40.6)	*G. lamblia* (30.1)
	Kaplan *et al*., 2011^78^	368	RV (49.5)	Norovirus (11.4)	
Kuwait	Albert *et al*., 2016^81^	109	Campylobacter (5.5)	*Salmonella* (2.8)	*C. difficile* (2.8)
Morocco	Benmessaoud *et al*., 2015^95^	122	*E. coli* (58.2)	RV (17.2)	*Shigella* (6.5)
Saudi Arabia	El-Sheikh *et al*., 2001^127^	576	RV (34.6)	*E. coli* (13.0)	EPEC (3.8)
	Johargy *et al*., 2010^134^	270	RV (22.2)	Adenovirus (7.4)	Astrovirus (3.7)
Yemen	Kirby *et al*., 2011^171^	290	RV (26.9)	Norovirus (10.3)	

AGE, acute gastroenteritis; DEC: diarrheagenic *E. Coli*; EAEC, enteroaggregative *E. Coli*; EPEC, enteropathogenic *E. coli*; ETEC, enterotoxigenic *E. coli*; RV, Rotavirus; WHO-EMRO, World Health Organization – Eastern Mediterranean Regional Office.

### RVGE seasonality patterns

A seasonality pattern of RVGE was reported in 95 of the 165 (57.6%) reviewed studies. Most studies (66/95, 69.5%) reported a peak of RVGE in the cold season from November to April. However, some studies also showed exceptional RVGE peaks occurring in late summer.^22,111,154,172^ Studies from Iran, Bahrain, and Saudi Arabia did not observe any seasonal variation of RVGE.^13,39,132^

### Detection techniques used

A total of 126 out of 147 studies that described RVGE prevalence also reported the isolation technique used for RV detection: 97 studies (77%) used enzyme immunoassay (EIA), 12 (9.5%) used latex agglutination (LA), 25 (19.8%) used polymerase chain reaction (PCR), 4 (3.2%) used polyacrylamide gel electrophoresis (PAGE) techniques, and a comparison with electron microscopy (EM) was made in 2 studies. Some of the studies also compared detection rates based on which techniques had been used. In Egypt, the positivity of RV detection varied between 34% and 39.5% according to LA and EIA techniques, respectively.^16^ In Jordan, the sensitivity of EM and EIA RV detection techniques were 18.9% vs. 39.6%, respectively.^72^ In Egypt, the sensitivity was higher when PCR (76.9%) was used as compared to EIA (67.7%).^30^

### Nosocomial RV infection

Nosocomial infection due to RV is also common. A study in Iraq showed that 32.4% of hospitalized children had nosocomial infections and 18.5% of them were due to RV.^41^ The nosocomial infection rate was 33.3% in Morocco,^92^ and in three different studies from Iran, the reported rates were 26.3%, 18.5% and 30%.^38,41,54^

### Disease severity and mortality rates

In Morocco, 1 child per 32 RV-infected children was hospitalized and 1 child per 389 RV-infected children died due to RVGE in a group of children under 5 years of age.^91^ Another Moroccan study reported that 30% of hospitalizations for diarrhea were due to RVGE, with a mortality rate of 40–50%.^90^ In Pakistan, 1 child per 40 infected children under 5 years of age had an episode of severe RVGE per year.^117^ A meta-analysis spanning from 1989 to 2004 estimated 19,933 deaths due to RVGE in Pakistan, constituting 3.8% of the global RVGE mortality.^109^ In Iran, 2,700 deaths due to RVGE occurred per year; it was estimated that 13–40% of all cases of diarrhea were due to RV and that 30% of them required hospitalization.^37,40,47^

### RV genotype and evolution of genotypes post-vaccine introduction

The genotype distribution of RV isolates in different studies was also reviewed. We evaluated the genotype combinations, where both G and P capsid protein combinations are reported. Studies conducted in 13 WHO-EMRO region countries (Bahrain, Egypt, Iran, Iraq, Jordan, Libya, Lebanon, Morocco, Oman, Pakistan, Saudi Arabia, Tunisia, and Yemen) reported RV genotype distribution (Table 3). Studies from Afghanistan, Palestine, Qatar, Somalia, and Sudan did not report on genotype distribution; Kuwait and UAE reported only G- or P-type, and were therefore not included in this evaluation. Overall, the genotype distribution was diverse across the region.

**Table 3.** RV genotype distribution (%) in WHO-EMRO countries.

Country	Study	G1P[8]	G1P[4]	G1P[6]	G2P[4]	G2P[8]	G3P[4]	G3P[8]	G3P[9]	G4P[6]	G4P[8]	G8P[9]	G9P[3]	G9P[6]	G9P[8]	G12P[6]	G12P[8]	Mix	Nt
Iran	Khalili *et al*., 2004^34^	95																	15
	Farahtaj *et al*., 2007^37^	59.2													15.5				
	Eesteghamati *et al*., 2009^40^	10.9			5.5						30.9								
	Modaress *et al*., 2011^45^	53.4	9.2															3.1	
	Kargar *et al*., 2012^46^																	60	12.5
	Shoja *et al*., 2013^48^	50			4.3			0.2	0.2		9.3				2.4		0.2	1.5	28
	Azaran *et al*., 2016^57^	80			20														13.7
	Azaran *et al*., 2018^60^	9.4			18.8	3.1		3.1			9.4				28.5		9.4	6.3	3.1
Saudi Arabia	Kheyami *et al*., 2008^130^	44			20			4							11		4		
	Kheyami *et al*., 2008^131^	89																	
	Tayeb *et al*., 2008^132^	60.7		3.5	1.7			3.5						10.7	16				
	Khalil *et al*., 2015^138^	49.3			1.5	0.7					0.7				9.6				
	Aly *et al*., 2015^139^	61.9	0.9					4.4							16.8	0.9	6.2		
	Al-Ayed *et al*., 2017^141^	46.2		1.3	25.6	2.6		3.8			1.3				10.3		5.1		3.8
Tunisia	Bouanane, 2011^148^	16.2						58.8		4.4	14.7							5.9	
	Trabelsi *et al*., 2000^150^	50																	
	Chouikha *et al*., 2007^153^	34	10	14	3		3	4		8	2				2			16	
	Trabelsi *et al*., 2010^157^	35.7		21.4	4.8		4.8	2.3		4.8	2.3							19.1	
	Sdiri-Loulizi *et al*., 2011^158^	23.9						45.6											
	Hassine-Zaafrane *et al*., 2011^159^	37.5			16.7			25			12.5				1.7				
	Chouikha *et al*., 2011^161^	12.2			25.5			25.5		2.5	1.8				2.2			12.6	
	Soltani *et al*., 2012^162^	6.5						24.4			13.3								
	Ben Salem-Ben Nejma *et al*., 2014^163^	6.4			12.9			10.5			2.4								1.6
	Soltani *et al*., 2015^164^							33.3			36.7								
	Ayouni *et al*., 2015^165^	21.9						25							37.5				
	Moussa *et al*., 2016^166^	34.4			4			16.3			8.9				10.3	2.6	1.9		
Pakistan	Qazi *et al*., 2009^117^	13	8.4		6	3.6				6	2.4			3.6	15			2.4	25
	Iftikhar *et al*., 2012^110^	25.3		21.1				1.5										1	
	Alam *et al*., 2013^111^	2.3	6.8	20.5	47.7	2.3									15.9				
	Tamim *et al*., 2013^112^	24.3		12.1	24.3									5.4	10.8	6.7		6.7	
	Kazi *et al*., 2014^118^	11.6		5.4	10.4					10.1				4.0	3				30.3
	Umair *et al*., 2017^115^	14.5	3.8	5.3	15.2	0.8	4.5	17.6						2.2	5.3	16.7	1.5	6.1	
Jordan	Kaplan *et al*., 2011^78^	56	1		14			1			4			5	13			3	
	Salem *et al*., 2011^79^	69	2.0		2.4			1.2			0.4			0.4	8.8				2.8
Iraq	Ahmed *et al*., 2006^69^	33		11	15						21			2	11			6	
Egypt	Naficy *et al*., 1999^19^	9	2		67													7	9
	Kamel *et al*., 2009^23^	17.1			40.8														
	Matson *et al*., 2010^24^	56.0	1.6	0.8	23.5	0.4	0.4				2.9			3.7	1.6				
	Ahmed *et al*., 2014^27^	18			13	2		2						1	12	1		2	42
	Shoeib *et al*., 2015^29^	19.5		3.9				37.7						1.3		2.6			
	Saudy *et al*., 2017^32^	26.7	13.3	2.2			4.4	15.6						6.7	20			8.9	2.2
Morocco	Benhafid *et al*., 2009^90^	33		3.7		0.7									30.5			20.9	
	Aghoutane *et al*., 2012^98^	55		2											12				
	Benhafid *et al*., 2013^93^	55	0.2	0.9	9.1	0.2	0.2	0.4			0.9			0.2	11.3			15	
	El Qazoui *et al*., 2014^94^	51.7		2.3	10.1	4.5					3.4				3.4			22.5	2.1
	Benmessaoud *et al*., 2015^95^	76.2							19			1.5							
	Benhafid *et al*., 2015^96^	57			15			0.3			0.6				7.8			11	
Lebanon	Dbaibo *et al*., 2013^84^										36.9								
	Ali *et al*., 2016^83^	36			17.8						15.9				26.4				
Libya	Abugalia *et al*., 2011^88^	27.8			1.7				0.6		2.8				65.9			0.6	0.6
Bahrain	Musawi *et al*., 2013^15^	58.8			11.8	11.8									11.8				
Oman	Al Awaidy *et al*., 2009^100^	3	3		25								4						16
	Al Baqlani *et al*., 2010^101^	19.1		1.8	8.2	0.9		13.6		0.9									
	Al Baqlani *et al*., 2016^102^	41.7			21.7			6.7							13.3			8.3	
Yemen	Kirby *et al*., 2011^171^	55			12										21		1		1
	Al-Badani *et al*., 2014^172^	15			55														
	Banajeh *et al*., 2015^173^ Pre-vac.	45.5			76.5														
	Banajeh *et al*., 2015^173^ Post-vac.	87.5			0.2														
	Al-Kamarany *et al*., 2016^174^ Pre-vac.	15			55														
	Al-Kamarany *et al*., 2016^174^ Post-vac.	31													27.5				

Nt, Non-typable; RV, Rotavirus; vac., vaccination; WHO-EMRO, World Health Organization – Eastern Mediterranean Regional Office.

G1P[8] was the most prevalent genotype combination in 7 of the 13 countries (i.e. Iran, Saudi Arabia, Jordan, Bahrain, Morocco, Iraq) for which data from the past 15 years are available (Table 3).^15,34,37,45,48,57,69,78,79,90,93–96,98,100–102,130–132,138,139,141^

G1P[8] and G2P[4] were the most common genotypes in Yemen and Egypt.^19,23,24,27,29,32,83,84,171,172^ In Lebanon, the genotype distribution varied over time and geographical location: the G4P[8] type disappeared from the northern part of the country in two out of the three studied seasons, while the G2P[4] genotype did not.^83^ In Oman, the distribution pattern of the G2P[4] and G1P[8] RV genotypes also varied with time.^100–102^

In Tunisia, both G1P[8] and G3P[8] were common, followed by G4P[8].^148,158,159,162–164,166^ RV genotype G9 was first detected in 2004 in Tunisia and from 2010, there was an increase in the prevalence of related genotypes (10.3–37.5%).^165,166^

In Pakistan, the distribution of genotype combinations was heterogeneous^110–112,117,118^ and G12P[6] was found to be one of the most prevalent RV genotype in 2014.^115^

The introduction of RV vaccines had a substantial effect on G1P[8] detection. In Saudi Arabia, a decrease was noted in G1P[8] detection from 51% to 37.1% after the vaccine was introduced, but G2P[4] increased from 21.6% to 33.3%.^141^

In Morocco, a periodic decrease in the detection of G1P[8] genotype was reported: 57% one-year post-vaccination and 54.7% two years post-vaccination.^96^ However, G2P4 was detected in 15% of samples in the pre-vaccination period and in 54% and 33% of samples, respectively, in the second and third years following the vaccine introduction. An increase was also seen for G9P[8] (7.8% in the pre-vaccination period, 16.6% and 67% in the second and third year of the post-vaccination period, respectively).

In Yemen, the G1P[8] detection rate increased from 45.5% to 87.5% and the G2P[4] detection rate decreased from 76.5% to 0.2% after vaccine implementation.^173^ In another study in Yemen, the G1P[8] detection rate increased from 15% in the pre-vaccination period to 31% in the post-vaccination period and became the predominant genotype, followed by G9P[8] (27.5%).^174^

In Iran, there was also an emergence of new RV genotypes over time, with G12P[8] being detected for the first time in 2016.^57^ In Egypt, genotype G6P[14] was first reported in 2011.^26^

Like in other parts of the world, mixed or non-typeable RV strains have also been detected in the WHO-EMRO region. In Iran, mixed genotype combinations, including G1P[4] and G1P[8], were detected in two studies, and the rate of mixed genotypes varied from 2.7% to 33.3%.^47,49^ Non-typed (Nt) strain detection in Iran ranged from 5% to 40.9%.^34,68^ In Pakistan, Egypt and Tunisia, the detection rates for mixed RV genotypes were 6.7%, 7% and 5.9%, respectively.^19,113,148^

### Vaccination status

The demographic characteristics and the type of RV vaccine used in the WHO-EMRO countries are shown in Table 4. An RV vaccine was available in 19 (86.4%) of the 22 WHO-EMRO countries; either publicly (10/22), both publicly and privately (5/22), or only privately (4/22). No RV vaccine was available in three (13.6%) WHO-EMRO countries (Iran, Somalia, and Syria). Fifteen (72.7%) WHO-EMRO countries had introduced an RV vaccine in their NIP (Afghanistan, Bahrain, Djibouti, Iraq, Jordan, Kuwait, Libya, Morocco, Pakistan, Palestine, Qatar, Saudi Arabia, Sudan, UAE, and Yemen). Six (27.3%) of the 22 EMRO countries were eligible for GAVI support. (Table 4).

**Table 4.** Demographics and RV vaccination status of the 22 WHO-EMRO countries.

COUNTRY	Geographic region	World bank classification (2017)	Gavi Eligibility^a^	RV vac. in NIP	Year of vac. implementation	Availability of vac.(private/public)	Recommendation status	Reimbursement
Afghanistan^b^	Asia	LIC	Yes	Yes. In Jan 2018 GAVI supported Rotarix in NIP	2018	Public	Included in NIP in 2014 but not introduced due to political situation.^175^	
Bahrain	Middle East	HIC	No	Yes. Rotarix in NIP	2008	Public	Recommended	Yes
Djibouti	Middle East	LMIC	NIP through GAVI support	Yes. Rotarix in NIP	2014	Public	Recommended	
Egypt	Africa	LMIC	No	No		Private	MoH not included in NIP.	No
Iran, Islam Rep. Of	Asia	UMIC	No	No		No	Not recommended for NIP	
Iraq	Middle East	UMIC	No	Yes. Rotarix in NIP	2012	Public	Recommended	
Jordan	Middle East	LMIC	No	Yes. RotaTeq in NIP	2015	Public	Recommended	
Kuwait	Middle East	HIC	No	Yes. RotaTeq	2017	Private & Public	Recommended	
Lebanon	Middle East	UMIC	No	No		Private	No Recommendation	
Libya	Africa	UMIC	No	Yes. RotaTeq in NIP	2013	Public	Recommendation in NIP as mandatory vaccination^176^	
Morocco	Africa	LMIC	No	Yes. RotaTeq in NIP	2012	Private & Public	Recommended	Only by some private insurance
Oman	Middle East	HIC	No	No		Private	Not Recommended	Only by some private insurance
Pakistan	Asia	LMIC	Yes	Yes	2016	Public & Private	Recommended	No
Palestine, Occupied territory	Middle East	-	No	Yes, Rotarix^177^	2016	Rostropovich Vishneskaya Foundation and United Nations Relief and Works Agency helped support Palestinian MoH to introduce RV vaccination in public program		
Qatar	Middle East	HIC	No	Yes, Rotarix	2009	Public	Recommended	Yes
Saudi Arabia	Middle East	HIC	No	Yes, Rotarix	2013	Private & Public	Mandatory	Public: FOC; private: insured = covered, if not: parents pay
Somalia	Asia	LIC	Yes	No		No	A country in conflict – No vaccine recommendations	
Sudan, Republic of	Africa	LMIC	Yes	Yes, Rotarix	2011	Public	Recommended	
Syrian Arab Republic	Middle East	LMIC	No	No		No	No recommendation. Country currently in conflict	
Tunisia	Africa	LMIC	No	No		Private (since 2009)	Not recommended in NIP	Only by some private insurance
United Arab Emirates	Middle East	HIC	No	Yes, Rotarix	2014	Private & Public	Recommended^178^	Yes
Yemen	Middle East	LMIC	Yes	Yes, Rotarix	2012	Public	Recommended in NIP	

FOC, free of charge; GAVI, the vaccine alliance; HIC, high-income countries; LIC, low-income countries; LMIC, low-middle-income countries; MoH, Ministry of Health; NIP, national immunization programs; RV, rotavirus; UMIC, Upper-middle-income countries; vac., vaccination; WHO-EMRO, World Health Organization – Eastern Mediterranean Regional Office.

^a^GAVI-eligible countries in 2017, https://www.gavi.org/support/sustainability/countries-eligible-for-support/

^b^Literature search was conducted as of December 5, 2017. As of January 2018, Afghanistan introduced rotavirus vaccine.

## Discussion

This review evaluated the RV infection status in the WHO-EMRO region, identified reductions in RV prevalence and mortality following implementation of RV vaccination and described existing RV surveillance systems in the region.

In the WHO-EMRO region, the observed RVGE burden varied between countries. In general, a low RVGE prevalence (<30%) was observed in Egypt, Saudi Arabia, and Tunisia, and a high prevalence (>30%) was reported in Iran, Lebanon, Morocco, Jordan, and Oman.^8^ The annual proportion of RVGE among reported episodes of AGE in the under-five-years-of-age population in this region was 42%, and recent studies have estimated that about 65,000 children die each year from RV infections in the 22 WHO-EMRO countries.^9^ This is likely to be an underestimation of the true prevalence, due to inadequate surveillance systems and lack of routine RV testing. Although a majority (81.8%) of EMRO countries have implemented RV vaccination, seven countries (Egypt, Iran, Lebanon, Oman, Somalia, Syria, and Tunisia) have not yet implemented RV vaccination in their NIPs. Furthermore, no RV vaccine is available in three (13.6%) EMRO countries (Iran, Somalia, and Syria).

RV was the most common AGE agent in the WHO-EMRO region in 76.3% of the reviewed studies. Our review also revealed that there is a wide variation (>20% difference between studies) in RV isolation rates in different studies conducted within the same countries; for example, in Iran,^40^ Pakistan,^105,114^ Saudi Arabia^132,135^ and Morocco,^89,95^ the difference of reported isolation rates was wide. However, in countries such as Tunisia^156,163^ and Jordan,^74,78^ the rate differences were smaller. Differences in study design, patient selection, and case definition could contribute to explain differences in observed incidences across studies.

This review also confirmed that as in Western Europe,^179^ G1P[8] and G2P[4] are the predominant genotypes circulating in the WHO-EMRO region and that available RV vaccines can provide protection against them. It is apparent that the circulating RV strain distribution regularly changes, as well as the dominant genotype, even in the absence of vaccines. Interestingly, G12P[8], a recently emerging serotype previously detected in Europe, Asia, and the Americas, has also been reported in some studies in Saudi Arabia.^8,139,141^ Unusual genotypes such as G1P[6], G2P[6], G3P[9], G4P[6], G9P[6] and G9P[8] have been detected in different countries,^118,132,148,157^ including the recent emergence of genotype G12P[6] in Pakistan^115^ and of genotype G12P[8] in Iran.^60^ Data of genotyping diversity from the WHO-EMRO region confirm that these findings are not specific to a particular geographic location but a natural phenomenon worldwide.

Mixed genotype combinations were detected in Iran,^49,60^ Tunisia,^149,150^ and Pakistan.^112^ These results are similar to recent findings in India, Indonesia, and Vietnam and are likely due to reassortments.^10^ The proportion of non-typeable and partially typeable genotype combinations also varied widely across countries.^6,48,49,100^ Differences in laboratory techniques, as well as different geographic settings, can explain these findings. With the use of more appropriate genotyping primers and advanced molecular techniques, the proportion of non-typeable strains has decreased in recent studies.

A shift in genotype predominance and circulation before and after vaccine introduction is not unusual and cannot be attributed to available vaccines. Countries such as Australia and Brazil that have used different vaccines have also faced the re-emergence of G2 strains after vaccine introduction.^180,181^ These data simply reflect the normal fluctuation in RV genotype frequency. In order to substantiate any vaccine pressure on genotype shift, continued surveillance over time, detailed phylogenic analysis, and full RV genome sequencing are required. Although these measures are currently not in place in the WHO-EMRO region, policy measures aimed at implementing these measures would greatly enhance our understanding of changes in RV strain circulation in the region.

In general, RV infections peak in the cooler winter months, and this pattern is similar for all WHO-EMRO countries except Gulf region countries. Studies from Saudi Arabia and neighboring countries showed no distinct peak, as RV disease was observed throughout the year with occasional peaks irrespective of season.^132^

Several countries that have implemented routine childhood vaccination against RV have documented a dramatic impact on severe diarrhea and RVGE requiring hospitalization.^182–185^ In the WHO-EMRO region, a small number of studies also show the effectiveness of vaccination on RVGE hospitalization, as well as RV detection in AGE cases. The results of these studies reflect that vaccination can markedly reduce RVGE disease burden, especially hospitalization due to diarrhea.^99,141,173,174^

The WHO recommends sentinel surveillance before and after the introduction of vaccines to monitor vaccine impact. As financial resources may be limited in this region, regional governments should consider sharing their data with local laboratories or existing surveillance networks to help achieve this goal. Documentation of the burden of disease and of circulating strains is essential for decision-making on vaccine introduction and vaccine effectiveness assessments. The burden of RVGE and the circulation of different RV strains should be monitored in a surveillance system and through standardized methods, which would enable comparisons between countries.

We observed that no single standard technique was used to isolate and genotype RV from stool samples. This is likely to have led to a variation of estimates, not only in different countries but also across studies conducted within a country. Thus, we decided to present descriptive results instead of direct comparisons.

The various studies reported here included patients from different age strata in both in- and outpatient settings, which may potentially lead to misclassifications. It is easily conceivable that patients admitted as inpatients were suffering from more severe disease and were therefore more likely to yield a pathogen from their stool sample than outpatient stool samples originating from patients with less severe infections. This was evident from studies in Saudi Arabia,^127^ where the RV detection rate was 5.9% in outpatient settings compared with 34.6% in inpatient settings; in Iran^66^ the rates were 20.9% and 79.1% in outpatient and inpatient settings, respectively.

Variations in the estimates between and within countries could also be due to different study populations, study time periods, RV isolation and detection techniques, and the definition of RVGE used as study inclusion criteria. In some countries such as Afghanistan, Bahrain, Iraq, Kuwait, Lebanon, Palestine, Qatar, Somalia, and Sudan, the number of studies and subjects enrolled was not adequate for accurately estimating the burden and strain distribution of RVGE.

Although our review is one of the first to explore the prevalence of RV in the WHO-EMRO region, it has several limitations. Firstly, there is also a possible bias in identifying a denominator for the estimates. In some studies, RV rates were detected over total stool samples collected, while in others, the RV proportion was reported among the cases where at least one pathogen was detected. Secondly, our search was limited to published studies with abstracts in English and French, which may have resulted in the exclusion of studies published only in local languages. Thirdly, gaps in reporting from several WHO-EMRO countries may be due to political instability and not a lack of RV infections, and political instability may also prevent the successful implementation of vaccination programs and longitudinal RV vaccine studies.

In conclusion, this review of 165 studies from WHO-EMRO countries revealed that RV was strongly associated with young age in children and remained the dominant etiology of diarrhea requiring hospitalization, even in countries that had recently introduced RV vaccination. The collated data in this study will serve as useful baseline RV epidemiological data for the planning and implementation of vaccination programs in the region. Although the burden of RVGE is likely to decrease following the successful introduction of RV vaccination programs, as exemplified by Yemen,^171^ ongoing surveillance is needed to determine the residual burden of RV, the genotype distribution of RV, and to monitor the evolving etiology of diarrhea in these countries. Data compiled in this report highlights that nearly all serious infections due to diarrhea occurred in children under 2 years of age, which stresses the need for protection early in life, and advocates for RV vaccine schedule completion by 12 months of age.

## Methods

The inclusion criteria for the search was any publication in English or French in a country of the WHO-EMRO region on RV diarrhea in children less than five years of age during the period of January 1990 to December 5, 2017. We used the keywords “rotavirus” and “[country name]” (for each individual country out of the 22 included in the WHO-EMRO) to search in The National Library of Medicine’s PubMed and grey literature (Figure 2). We also included publications in local medical journals and in local languages for which abstracts in English or in French were available. We further reviewed the cross-referenced articles from the retrieved ones.

A total of 426 published articles were identified, 387 from PubMed and 39 from local journals. Articles studying animals or environment (97), reporting the clinical aspects of infection studies (116), studying vaccine implementation or cost-effectiveness (32), or using non-standard detection techniques (16) were excluded, and 165 articles were included for complete full-text review and qualitative assessment (Figure 2).

Authors, year of publication, type of article, patient type and numbers were reported for each publication. The prevalence of RV infection mentioned in the articles was the main focus of this study, and we also retained articles mentioning RVGE mortality, or RV genotyping. In addition, and where available, data on nosocomial infections, seasonality, techniques used for the detection, RV genotype distribution, variation over time in the proportion of RVGE, and evolution of genotype distribution, presence of other enteric co-pathogens and the vaccination status of the country was also abstracted. Each datum was abstracted by one author, and crossed-check by another author. Next, we calculated the RVGE prevalence as the number of reported RVGE cases over the total number of samples included in each study, to use a standard measure of the RVGE prevalence. When possible (i.e. when three studies or more were retrieved for one country), the mean and median prevalence was given for each country. Data were stratified by country.

## Trademark statement

*Rotarix* is a trademark of the GSK group of companies. *Rotateq* is a trademark of Merck and Co Inc.
