# Supplementary material for: Systematic review of the rotavirus infection burden in the WHO-EMRO region
Source: Hum Vaccin Immunother. 2019 May 17;15(11):2754–68. doi: 10.1080/21645515.2019.1603984 (PMC6930073; doi:10.1080/21645515.2019.1603984)
Supplement: Supplemental Material [file khvi-15-11-1603984-s001.docx]

**Supplemental material**

## **Table S1:** RV surveillance data from the North African countries of the WHO-EMRO region

| Country (number of studies) |  | Authors, year | Study population, year | Number of pts | Age group (y) | RV detection technique | Season | RV prevalence (%) | RV (+) % / age group |
| --- | --- | --- | --- | --- | --- | --- | --- | --- | --- |
| Egypt (17) |  | Amer AA, 1990^16^ | Comparison of techniques | 200 |  | LA/EIA |  | 34-39.5 ^a^ |  |
|  |  | Pazzaglia G *et al*., 1993^17^ | Out + In | 880 | Mean: 10 months |  |  | 28.6 |  |
|  |  | Radwan SF, 1997^18^ | In | 129 pts + 51 ctr.: 180 | <1 | EIA | August-December | 35.6 ^b^ |  |
|  |  | Naficy AB *et al*., 1999^19^ | Out (population- based cohort) | 178 | <3 | EIA | August-September | 25.8 | High: 6-11m |
|  |  | Holmes JL *et al*., 1999^20^ | Unusual G8 strains | longitudinal survey | <2 |  |  |  |  |
|  |  | El-Mohamady H *et al*., 2006^21^ | Out (diarrhea) | 253 | <5 |  |  | 21.3 |  |
|  |  | Wierzba TF *et al*., 2006^22^ | Out, 2000/2 | 1,026 | <6 |  | Late summer | 25.3 | 89% in <2y |
|  |  | Kamel AH *et al*., 2009^23^ | Out, 2006/7 | 230 | <18 | EIA | Winter (88.7%) | 33 |  |
|  |  | Matson DO *et al*., 2010^24^ | In (Hospital-based surveillance) | 1,026 | <5 | EIA |  | 25.2 |  |
|  |  | Kamel AH *et al*., 2010^25^ | Out, 2006/7 (+ sewage) | 226 |  |  | November-February | 33.6 |  |
|  |  | El Sherif, 2011^26^ | 1st detection of G6P14 type |  |  |  |  |  |  |
|  |  | Ahmed SF *et al*., 2014^27^ | Out, 2004/7 | 348 | <2 | EIA | September- November | 40.2 |  |
|  |  | El Senousy WM *et al*., 2015^28^ | In; 2011/12 (+ envir.) | 110 | <5 | PCR | October- November | 54.5 |  |
|  |  | Shoeib ARS *et al*., 2015^29^ | Out + In | 197 (67 + 130) |  |  | Winter | 39.1 (Out: 29.9%; In: 43.9%) | High in <1y |
|  |  | Ibrahim AB *et al*., 2015^30^ | In, 2014/15 (comparison of techniques) | 65 | <5 | EIA/PCR |  | 76.9 |  |
|  |  | El-Shabrawi M *et al*., 2015^31^ | In; 2007/9 | 356 | <5 |  | May-October | 11 |  |
|  |  | Saudy N *et al*., 2017^32^ | In; 2010/12 | 92 | <3 | PCR | Autumn-winter | 48.9 | 73.3 % in <1y |
| Morocco (9) |  | Benhafid M *et al*., 2009^90^ | In; 2006/7 | 314 | <5 | EIA | September-January | 44 | 76% in <1y |
|  |  | Aouad FZ, 2011^97^ | In; 2009/10 | 88 |  |  | October-December | 37.5 | . |
|  |  | Aghoutane M, 2012^98^ | Out; 2006/10 | 2,526 |  |  |  | 39 | 69% in <1y |
|  |  | Benhafid M *et al*., 2012^91^ | In; 2006/10 | 1,841 | <5 | EIA | October-December | 40.2 | 48.7% in <1y |
|  |  | Bentama I *et al*., 2012^92^ | Nosocomial RV inf. | 36 (26 preterm) |  |  |  | 33.3 |  |
|  |  | Benhafid M, 2013^93^ | In; 2006/9 | 1,388 |  | EIA | October-November | 42 | 64% in <1y |
|  |  | El-Qazoui M *et al*. 2014^94^ | In; 2011 | 335 | <5 | EIA | January | 26.6 | 36% in <1y |
|  |  | Benmessaoud R *et al*., 2015^95^ | In; 2011/12 | 122 | <5 | PCR | January-March | 17.2 |  |
|  |  | Benhafid M *et al*., 2015^96^ | In; pre-vac. date, 2006/10 | 1,861 |  |  | November | 41 |  |
|  |  | Benhafid M *et al*., 2015^96^ | In; post-vac. date, 2011/13 | 533 |  |  | November/ February | 24 ^c^ |  |
| Tunisia (20) |  | Bouanane I, 2011^148^ | In; 2011 |  |  |  |  | Genotyping |  |
|  |  | Moalla H *et al*., 1994^149^ | Out; 1986 | 170 | <6 |  |  | 12.4 |  |
|  |  | Trabelsi A *et al*., 2000^150^ | In; 1995/9 | 375 | <5 | PAGE | November-February | 17.3 |  |
|  |  | Fodha I *et al*., 2006^151^ | Out + In; 2003/5 | 638 (257 + 381) | <5 | EIA/PAGE | November-March | 20.8 |  |
|  |  | Al-Gallas N *et al*., 2007^152^ | Out; 2001/4 | 115 (child.), 73 (ad.) |  |  | Autumn | 6.1 (child), 1.4 (adult) |  |
|  |  | Chouikha A *et al*., 2007^153^ | Out + In; 1995/2004 | 982 (354 + 628) | <5 | EIA/PAGE |  | 22 (Out: 18; In: 25) |  |
|  |  | Sdiri-Loulizi K *et al*., 2008^154^ | Out + In; 2003/5 | 632 (380 + 252) | <12 |  | Winter, June -September | 22.5 | High in <2y |
|  |  | Tinsa F *et al*., 2009^155^ | In; 2007 | 117 | <5 | EIA | January; May; September | 25.6 |  |
|  |  | Chouikha A *et al*., 2009^156^ | In, 2003/5 | 638 | <5 | EIA |  | 20.8 |  |
|  |  | Trabelsi A *et al*., 2010^157^ | Out + In; 2000/3 | 309 | <5 | EIA/LA | October-March | 26.2 |  |
|  |  | Sdiri-Loulizi K *et al*., 2011^158^ | Out + In; 2003/7 | 788 | <12 | PCR | June-September | 27 (Out: 13; In:29) |  |
|  |  | Hassine-Zaafrane M *et al*, 2011^159^ | Out + In; 2007/10 | 435 (197 + 238) | <13 | EIA/PCR | January-February | 28.3 (28.9 and 26.5) | 85% in <3y |
|  |  | Chouikha A *et al*., 2011^160^ | Out + In; 2005/7 | 1,503 | <5 | EIA |  | 21.0 (2005: 21.3; 2006: 17.4; 2007: 26.2) |  |
|  |  | Chouikha A *et al*., 2011^161^ | Electrotyping | 278 RV (+) | | PAGE |  |  |  |
|  |  | Soltani M *et al*., 2012^162^ | In; 2009/11 | 621 | <5 | EIA/PCR | Winter | 30.3 | 93.3% in <2y |
|  |  | Ben Salem-Ben Nejma I *et al*., 2014^163^ | Out; 2008/9 | 124 | <5 | EIA | Autumn-Winter | 33.9 |  |
|  |  | Soltani M *et al*., 2015^164^ | In; 2009/11 | 279 | <5 |  | December-February | 23.3 |  |
|  |  | Ayouni S *et al*., 2015^165^ | In; 2011/12 | 114 | <6 | EIA/PCR | Cold season | 28.1 | 80% in <14m |
|  |  | Moussa A *et al*., 2016^166^ | Out + In; 2009/14 | 1,127 (167 + 960) | <5 | EIA/PCR | November-March | 24 (Out: 18.5%, In: 24.9%) |  |
|  |  | Moussa A *et al*., 2017^167^ | Out + In; 2009/14 | 1,127 | <5 |  |  | 24 |  |
| Somalia (2) |  | Gargano LM *et al*., 2015^142^ | Vac. 2012 | 606,917 | <1 |  |  |  | ^d^ |
|  |  | Ope M *et al*., 2017^143^ | In; refugee camp; 2014  (Diagnostic test comparison) | 213 | <5 | EIA |  | 33.3 |  |
| Sudan (4) |  | Elhag WI *et al*., 2013^144^ | Out; diarrhea | 710 (<5:330, >5: 182, A: 198) | Adults + Children (<5: 46%) | ICT/PCR |  | 11.7 |  |
|  |  | Mustafa A *et al*., 2014^145^ | In; 2009/11 | 10,910 | <5 | EIA | March-May + November-December | 36 | 91% ^e^ in <2y |
|  |  | Magzoub MA *et al*., 2013^146^ | In; 2009/11 | 755 | <5 | EIA |  | 16 | 75.2% in <1y |
|  |  | Saeed A *et al*., 2015^147^ | Out; diarrhea | 437 | <5 | EIA | Winter | 22 |  |
| Libya (5) |  | Kalaf RN *et al*., 2011^85^ | In; 2008/9 | 200 | <5 | EIA | December-March | 33 | 36.1% in <2y |
|  |  | Rahouma A *et al*., 2011^86^ | Out; 2008 | 239 | <5 | EIA |  | 13.4 |  |
|  |  | Alkoshi S *et al*., 2014^87^ | Out + In; 2012/13 | 545 (135 + 410) | <5 | EIA | February | 57 (Out: 53%, In: 58%) | 86% in <2y |
|  |  | Abugalia M *et al*., 2011^88^ | Out + In; 2007/8 | 520 (260 + 260) | <5 | EIA/PCR | January | 31.5 (Out: 27,3%, In: 35.8%) | 65.2% in <1y |
|  |  | Alkoshi S *et al*., 2015^89^ | In; 2013 | 410 | <5 | EIA | December-March | 58 | 86% in <2y |

^a^ depending on the technique used

^b^ in total, patients and controls

^c^ from 41% to 24%: 41.5 % reduction

^d^ Children <1: RVGE: 3,864 deaths, 606,917 cases/2012

^e^ hospitalizations

ad., adult; child.: children; ctr., control; EIA, enzyme immunoassay; ICT, Immunochromatography; In, inpatient; LA, latex agglutination; m, months; Out, outpatient; PAGE, polyacrylamide gel electrophoresis; PCR, polymerase chain reaction; pts, patients; RV, rotavirus; vac., vaccination; WHO-EMRO, World Health Organization - Eastern Mediterranean Regional Office; y, years.

## **Table S2:** RV surveillance data from the Middle East countries of the WHO-EMRO region.

| Country (number of studies) |  | Authors, year | Study population, year | Number of pts | Age group (y) | RV detection technique | Season | RV prevalence (%) | RV (+) % / age group |
| --- | --- | --- | --- | --- | --- | --- | --- | --- | --- |
| Saudi Arabia (21) |  | El Assouli SM *et al*., 1992^121^ | In; 1988/9 | 363 | Infants, young children | EIA | January-February | 46 | 87.4% in <2y |
|  |  | Mohammed KA *et al*., 1994^122^ | In; 1988/92 | 1,242 | <5 | EIA | Winter | 42.2 |  |
|  |  | Milaat WA *et al*., 1995^123^ | In | 1,726 | <5 | EIA | Winter (Jeddah), Summer (Al-taif) | 41.3 | High in 6-14m |
|  |  | El Assouli SM *et al*., 1995^124^ | In; 1988/92 | 1,242 |  | EIA |  | 42.2 |  |
|  |  | El Assouli SM *et al*., 1996^125^ | Out; (Diarrheal cases) | 349 |  | EIA |  | 43 | 87% in <2y |
|  |  | El-Assouli SM, *et al*., 1996^126^ | Electropherotyping study | 523 (+) for RV |  |  |  |  |  |
|  |  | El-Sheikh SM *et al*., 2001^127^ | Out + In; 1995/6 | 576 | <5 |  |  | Out: 5.9; In: 34.6 |  |
|  |  | Ghazi HO *et al*., 2005^128^ | In (comparison of techniques) | 479 | Infants, young children | LA/EIA |  | 10 | high in <1y |
|  |  | Kheyami AM *et al*., 2006^129^ | Review, (22 studies: 1982-2003) |  |  |  | Difference of seasonality | 10-46 (median 30) | high in <2y |
|  |  | Kheyami AM *et al*., 2008^130^ | Out + In; 2004/5 | 984 (423 + 561) | <5 | EIA | November-February | 19 (Out: 7; In: 28) | 83% in <2y |
|  |  | Kheyami AM *et al*., 2008^131^ | In; 2004/5 | 454 |  |  |  | 12 | 24% in <5y |
|  |  | Tayeb HT *et al*., 2008^132^ | Out + In; diarrhea, 2002/4 | 1,000 | <6 | EIA | April | 6 | 61.6 % in <1y, 38.3% in >1y |
|  |  | Kheyami AM, 2010^133^ | Review (1982-2008) |  |  |  |  | median 30 ^a^ |  |
|  |  | Johargy A *et al*., 2010^134^ | Out; 2008 | 270 | <5 |  |  | 22.2 |  |
|  |  | Tayeb HT *et al*., 2011^135^ | In; 2008/10 | 1,007 |  | EIA | No seasonality peaks | 65.5 | 81% in <1y |
|  |  | Afifi R *et al*., 2013^136^ | In; 2010 | 301 | <14 | EIA | October-December | 42.9 | High in <2y |
|  |  | Abdel-Moneim AS *et al*., 2015^137^ | In (Sequence diversity for VP4/7) | 80 | <2 | Chromato |  | 15 |  |
|  |  | Khalil M *et al*., 2015^138^ | In; 2007/8 | 970 | <5 | EIA |  | 40.7 | 83.1% in <2y |
|  |  | Aly M *et al*., 2015^139^ | In; 2011/12 | 541 |  | EIA | October-November | 31.6 | 69.4% in <1y |
|  |  | Hegazi MA *et al*., 2017^140^ | In; 2015 | 359 | <5 | ICT |  | 3.9 |  |
|  |  | Al-Ayed MS *et al*., 2017^141^ | In; 2013/15 | 850 | <5 |  |  |  | ^b^ |
| Lebanon (3) |  | Al-Ali M *et al*., 2011^82^ | In; 2010 | 79 | <10 | ICT |  | 48.1 |  |
|  |  | Dbaibo G *et al*., 2013^84^ | In; 2007/8 | 487 | <5 |  | December-March | 27.9 |  |
|  |  | Ali Z *et al*., 2016^83^ | In; 2011/13 | 1,414 | <5 | PCR | December-January | 30.3^c^ |  |
| Bahrain (3) |  | Dutta SR *et al*., 1990^13^ | Out + In; 1984/6 | 698 (216 + 482) | <5 | EIA | No seasonal difference | 20.8  (Out: 6%, In: 27.4%) | 26.6% in <1y |
|  |  | Ismaeel AY *et al*., 2002^14^ | In; 1998/2000 | 653 | <3 | LA |  | 13.9 |  |
|  |  | Al Musawi M *et al*., 2013^15^ | In; 2006/7 | 239 | <5 | EIA | April | 44.8 | 76.6% in <2y |
| Oman (4) |  | Aithala G *et al*., 1996^99^ | In; 1992 | 217 | <5 | EIA | November-April | 31.3 |  |
|  |  | Al Awaidy SA *et al*., 2009^100^ | In; 2006/8 | 3,470 | <5 | EIA | December-May | 49.3 | 69% in <2y |
|  |  | Al Baqlani S *et al*., 2010^101^ | In; 2005 | 310 | <5 | EIA |  | 57.4 | 88.8% in <2y |
|  |  | Al Baqlani S *et al*., 2016^102^ | In; 2009/13 | 6,034 | <5 | EIA | December-April | 48.5 |  |
| Palestine (2) |  | Abu-Elamreen F *et al*., 2008^103^ | In; 2005 | 150 | <5 | PCR |  | 28.0 | 90% in <2y |
|  |  | Laham *et al*., 2015^104^ | In; 2011 | 96 | <5 | EIA |  | 3.1 | Higher in 3y |
| Qatar (1) |  | Al-Thani A *et al*., 2013^120^ | In; 2009 | 288 | Children + adults | PCR | June-September | 10.4 | 70% in <10y |
| UAE (3) |  | Ijaz MK *et al*., 1994^168^ | In; 1990/2 | 650 | <10 | LA | December-May | 21.4 | 59.9% in <1y |
|  |  | Howidi MH *et al*., 2014^169^ | In; 2009/10 | 758 | <5 | EIA | February | 50.3 | 88.1 % in <3y |
|  |  | Cheriathu J *et al*., 2014 (abstract)^170^ | In; 2011/12 | 2,783 | <5 |  | January-April | 24.7 |  |
| Yemen (4) |  | Kirby A *et al*., 2011^171^ | Out + In; 2007/9 | 290 (151 + 139) | <5 | EIA/PCR |  | 26.9  (Out: 24%, In: 30%) |  |
|  |  | Al-Badani A *et al*., 2014^172^ | In; 2006/8 | 795 | <5 | EIA/PCR | May-June | 45.2 |  |
|  |  | Banajeh SM *et al*., 2015^173^ | Out + In; 2007/14 (pre-vac. 2007/11; post-vac. 2013/14) | 5,691 | <5 | EIA | October-December | 35.3 | 62.4% in <1y ^d^ |
|  |  | Al-Kamarany MA *et al*., 2016^174^ | In; 2009-12 | Pre-vac. Per.: 1,776 | <5 | EIA/PCR | December | 40.6 (2009: 43.8%; 2010: 37.4 %) |  |
|  |  | Al-Kamarany MA *et al*., 2016^174^ | 2013-14 | Post-vac. Per.: 1,114 | <5 | EIA/PCR | November | 14.1 (2013: 17.9 %, 2014: 10.5%) ^e^ |  |
| Jordan (9) |  | Nimri LF *et al*., 1996^71^ | Out; | 220 | <5 | EIA |  | 35.0 | 62% in <2y |
|  |  | Meqdam M *et al*., 1997^72^ | In; 1992/3 (comparison of techniques) | 439 | <3 | EIA/EM |  | 18.9-39.6 ^f^ |  |
|  |  | Youssef M *et al*., 2000^73^ | In; 1993/4 | 265 | <5 | EIA |  | 32.5 |  |
|  |  | Battikhi MN, 2002^74^ | Out; 1997/2000 | 1,400 |  |  |  | 26.6 |  |
|  |  | Nimri LF *et al*., 2004^75^ | Out; 1999/2001 (Acute/persistent diarrhea) | 143 | <10 | EIA |  | 40.6 |  |
|  |  | Khuri-Bulos N *et al*., 2006^76^ | In; Retrosp. study in 4 hospitals | 1,028 |  | EIA | May-August | 27.1 ^g^ |  |
|  |  | Nafi O, 2010^77^ | In; 2007/8 | 148 | <5 | EIA |  | 39.9 | >50% in <2y |
|  |  | Kaplan NM *et al*., 2011^78^ | In; 2006/7 | 368 | <5 | EIA |  | 49.5 |  |
|  |  | Salem K *et al*., 2011^79^ | In; 2007/8 | 698 | <4 | ICT | November-January | 35.5 | 88% in <2y |
| Iraq (3) |  | Ahmed HM *et al*., 2006^69^ | In; 2005 | 260 | <5 | EIA |  | 36.9 |  |
|  |  | Alrifai SB *et al*., 2009^41^ | Nosocomial diarrhea, 2004/5 | 84 | <5 |  |  | 18.5 |  |
|  |  | Ahmed S *et al*., 2013^70^ | In; 2008 | 976 | <5 | EIA |  | 40.4 |  |
| Kuwait (2) |  | Marmash RW *et al*., 2007^80^ | In; 2005/6 | 172 | <5 | EIA |  | 43.6 |  |
|  |  | Albert MJ *et al*., 2016^81^ | In; 2014/15 (Comparison of techniques) | 109 |  | Luminex/EIA |  | 10-20 ^f^ |  |

^a^ 12-18 for recent studies

^b^ Decrease from 12-46 to 9.2 during 2-year post- vaccination period

^c^ 18.1% of 428 + cases are vaccinated

^d^ Prevalence RVGE: 48 % decrease. Hospitalization: pre- vaccination: 42.9; post- vaccination: 18.5 (2014)

^e^ The incidence of RV diarrhea reduced by 75.9 %. Hospitalization due to RV diarrhea reduced by 75.9 % (from 43.8 % in 2009, to 10.5 % in 2014)

^f^ depending on the technique used

^g^ 1995: 25%; 2002: 35.2%; 2003: 24.7%; 2004: 25.8%

EIA, enzyme immunoassay; EM, electron microscopy; ICT, Immunochromatography; In, inpatient; LA, latex agglutination; m, months; Out, outpatient; PAGE, polyacrylamide gel electrophoresis; PCR, polymerase chain reaction; Per.: Period; RV, rotavirus; UAE, United Arab Emirate; vac: vaccination; WHO-EMRO, World Health Organization - Eastern Mediterranean Regional Office; y, years.

## **Table S3:** RV surveillance data from the Asian countries of the WHO-EMRO region

| Country (number of studies) |  | Authors, year | Study population, period | Nb of pts | Age group (y) | | RV detection technique | Season | RV prevalence (%) | RV (+) % / age group | |
| --- | --- | --- | --- | --- | --- | --- | --- | --- | --- | --- | --- |
| Iran (37) |  | Amini S *et al*., 1990^33^ | Out; 1986/7 | 915 | <5 | | PHA/LA | April-May | 25 | 28% in 7-24m | |
|  |  | Modarres S *et al*., 1995^61^ | Out + In; 1993/4 | 704 | <5 | | EIA | April-June | 15.3 (Out: 12.1; In: 21.9) | 89.8% in <2y | |
|  |  | Khalili B *et al*., 2004^34^ | Out + In; 2001/2 | 504 (245 + 259) | <5 | | EIA | November-February | 27.0 (Out: 18; In: 35) | 37% in <2y | |
|  |  | Zarnani AH *et al*., 2004^35^ | Out + In | 704 (233 + 471) | <5 | | EIA | March-April | 15.3 (Out: 12.1; In: 21.9) | 89.9% in <2y | |
|  |  | Shahrzad M, *et al*., 2005^62^ | In; 2000/4 | 1,250 | <5 | | LA | December-February | 28.4 | 70% in <2y | |
|  |  | Taremi M *et al*., 2005^63^ | In; 2003/4 | 370 | <5 | | EIA, PCR | Winter | 25.3 | 40.2% in <2y | |
|  |  | Samarbafzadeh A *et al*., 2005^36^ | Out + In; 2001/2 | 200 (137 + 63) | <2 | | PAGE |  | 29.5 (Out: 26.3; In: 36.5) | 40.2% in 7-12m | |
|  |  | Kazemi A *et al*., 2006^64^ | In; 2003/4 | 185 | <5 | | EIA | January | 30.8 | 84.2% in <2y | |
|  |  | Farahtaj F *et al*., 2007^37^ | Out + In pts; 2003/4 | 374 | <5 | | EIA | April-May/ September-November | 24.6 | peak in <2y | |
|  |  | Kordidarian R, 2007^38^ | Nosocomial; 2003/4 | 80 | <2 | | LA |  | 26.3 ^a^ |  | |
|  |  | Modarres S *et al*., 2008^39^ | In; 2002/5 | 1250 | <5 | | EIA/PAGE | peak in cool months | 32.3 |  | |
|  |  | Yahyapour Y *et al*., 2008^65^ | In; 2004/5 | 208 |  | | EIA | Winter-Autumn | 61.1 | 29.8% in <1y | |
|  |  | Eesteghamati A *et al*, 2009^40^ | In; 2006/7 | 2198 | <5 | | EIA | September-January | 59.1 | 85% in <2y | |
|  |  | Alrifai SB *et al*., 2009^41^ | Nosocomial; 2004/5 | 259 | <5 | | LA |  | 18.5 | 36.5% in <1y | |
|  |  | Zaraei-Mahmoodabadi B, 2009^66^ | Out + In; 2005/6 | 260 (67 + 193) | <5 | | EIA | Winter | 35 (Out: 20.9; In: 79.1) |  | |
|  |  | Sadeghian A, 2010^42^ | In; 2006/7 | 156 | <6 | | LA | Winter | 28.8 | 69% in <2y | |
|  |  | Hamkar R, 2010^43^ | In; 2005/6 | 400 | <10 | | EIA | Winter | 62.0 | 66.1% in <1y | |
|  |  | Ghorashi Z *et al*., 2011^44^ | In; 2007/9 | 511 | <3 | | EIA | Autumn | 55.6 |  | |
|  |  | Modaress S *et al*., 2011^45^ | In; 2004/8 | 700 | <5 | | PAGE |  | 18.7 |  | |
|  |  | Ataei-Pirkooh *et al*., 2011^67^ | In; 2009 | 100 | <6 | | LA |  | 43.0 | 39.5% in <1y | |
|  |  | Kangar M *et al*., 2011^68^ | In; 2009/10 | 316 | <5 | | EIA/PCR | Autumn | 27.8 | 79.5% in <2y | |
|  |  | Kargar M *et al*., 2012^46^ | In; 2007/8 | 141 | <5 | | EIA/PCR | Summer | 28.4 | 72.9 % in <2y | |
|  |  | Najafi A *et al*., 2012^47^ | In; 2007/8 | 138 | <5 | | EIA/PCR | Autumn | 34.8 |  | |
|  |  | Shoja Z *et al*., 2013^48^ | Molecular analysis | 10 (+) pts. For RV |  | | PCR |  |  |  | |
|  |  | Kargar M *et al*., 2012^49^ | In; 2009/10 | 163 | <5 | | EIA | Winter | 46.0 |  | |
|  |  | Motamedifar M *et al*., 2013^50^ | In; 2008/10 | 827 | <11 | | EIA | September-October | 42.0 | High in <3y | |
|  |  | Najafi A *et al*., 2013^51^ | In; 2008/10 | 375 | <7 | | EIA | November-February | 24.3 |  | |
|  |  | Shoja Z *et al*., 2013^48^ | Review of 19 papers | In | <5 | |  |  | 15.3-67.6 (Average: 36.5) | High in <2y | |
|  |  | Shokrollahi MR, 2014^52^ | In; 2009/11 | 80 |  | | ICT | Winter | 48.8 |  | |
|  |  | Moradi-Lakeh M *et al*., 2014^53^ | Review of 36 papers | 15,368 children |  | |  | Cold season (high) | 38.3 | High in <6y | |
|  |  | Khoshdel A *et al*., 2014^54^ | Nosocomial; 2010/11 | 100 | <5 | | PCR |  | 30 |  | |
|  |  | Kargar M *et al*., 2014^55^ | In; 2010/11 | 184 | <5 | | EIA | Autumn | 28.3 |  | |
|  |  | Sharifi-Rad J *et al*., 2015^56^ | In; 2011/12 | 82 | <1 | | ICT |  | 70.2 | High in <5m | |
|  |  | Azaran A, *et al*., 2016^57^ | In; 2011/12 | 200 | <5 | | EIA/PCR | December | 50 |  | |
|  |  | Mousavi Nasab SD *et al*., 2016^58^ | In; 2013/14 (RV + Norovirus Coinfection) | 170 (130 + 40) | <5 | | PCR | Winter | 28.8 ^b^ | | |
|  |  | Monavari SHR *et al*., 2017^59^ | Review of 43 papers |  | <6 | |  |  | 6.4-79.3 (Average: 39.9) | |  |
|  |  | Azaran A *et al*., 2018^60^ | In; 2015/6 | 100 | <5 | | LA |  | 32.0 | | High in 6-8m |
| Pakistan (15) |  | Mubashir M *et al*., 1990^105^ | Out; 1983/5 | 402 | <3 | | EIA |  | 8.2 | | High in <2y |
|  |  | Huilan S *et al*., 1991^106^ | Out; 1982/5 - 5 countries | 758 (Pakistan) | <3 | | EIA/EM |  | 14.0 | | 71% in <1y |
|  |  | Agboatwalla M *et al*., 1995^107^ | Out 1990/1 | 203 (1990), 119 (1991) | <5 | | EIA | September-February | 12.3 (1990); 24.4 (1991) | | Overall: 16.8 (calculated) |
|  |  | Shah Y *et al*., 1999^116^ | Out; 1995/6 | 210 | <2 | | EIA |  | 29 | | High in 13 – 24m |
|  |  | Nishio O *et al*., 2000^108^ | Out; 1990/7 | 818 |  | | EIA | November | 13.7 | |  |
|  |  | Qazi R *et al*., 2009^117^ | Out; in 2 communes | 575 | <5 | |  | Winter | 16.9 | | 64% in <1y |
|  |  | Parashar UD, 2009^109^ | Review of 76 studies |  |  |  | |  |  | | ^c^ |
|  |  | Afzal A *et al*., 2010^119^ | In; 2006/7 | 500 | <5 | | EIA |  | 29.4 | | 78.9% in <1y |
|  |  | Iftikhar T *et al*., 2012^110^ | In; 2010 | 300 | <5 | |  |  | 57.3 | | 63.4% in <1y |
|  |  | Alam MM *et al*., 2013^111^ | In; 2008/9 | 1,306 | <5 | |  | January-April / July-September | 34.2 | |  |
|  |  | Tamim S *et al*., 2013^112^ | In; 2010 | 311 |  | | EIA | August-September | 23.8 | | 53.9% in <10m |
|  |  | Habib MI *et al*., 2014^113^ | In; 2007 | 300 | <5 | |  |  | 62.7 | | 80.3% in <3y |
|  |  | Kazi AM *et al*., 2014^118^ | In; 2006/8 | 6,679 | <5 | | EIA | November-March | 30.5 | | 60.9% in <1y |
|  |  | Alam MM *et al*., 2015^114^ | In; 2009/10 | 563 | <5 | | EIA/PCR | May-July | 65.9 | | 70.0% in <1y |
|  |  | Umair M *et al*., 2017^115^ | In; 2014 | 502 | <5 | | EIA |  | 29.3 | |  |
| Afghanistan (1) |  | Elyan D *et al*., 2014^12^ | In; 2009/10 | 432 | <5 | | EIA | Winter | 76.2 | | 88.1% in <1y |

^a^ Nosocomial infection due to RV

^b^ Norovirus: 8.8; RV + +Norovirus: 3.5

^c^ 19,933 deaths with 3.8 % mortality rate

EIA, enzyme immunoassay; EM, electron microscopy; ICT, Immunochromatography; In, inpatient; LA, latex agglutination; m, months; Out, outpatient; PAGE, polyacrylamide gel electrophoresis; PHA: reversed passive hemagglutination; PCR, polymerase chain reaction; pts., patients; RV, rotavirus, World Health Organization - Eastern Mediterranean Regional Office; y, years; WHO-EMRO.
